# Supplementary material for: Collective gradient sensing in fish schools
Source: Sci Rep. 2018 May 15;8:7587. doi: 10.1038/s41598-018-26037-9 (PMC5954127; doi:10.1038/s41598-018-26037-9)
Supplement: Supplementary file 1 — Supplementary Information [file 41598_2018_26037_MOESM1_ESM.pdf]

Supplemental Information: Collective gradient sensing in fish schools

James G. Puckett, Aawaz R. Pokhrel, and Julia A. Giannini

Department of Physics, Gettysburg College,

Gettysburg, Pennsylvania 17325, USA

(Dated: 1st March 2018)

The two videos **M1shiner** and **M2tetra** present typical performance of groups of golden shiners and rummy nose tetras, respectively. The fish silhouettes are shown in orange and are overlaid on the noisy light field which the fish are trying to navigate. In both videos, the noise-scale is 0.25 and the group size is 32.

I. HUSBANDRY

We studied the gradient sensing performance of schools of golden shiners (*Notemigonus crysoleucas*) and rummy nose tetras (*Hemigrammus bleheri*) in a laboratory. While both are freshwater fish that naturally school in shallow water, shiners (a cyprinid found in cool waters of eastern North America) and tetras (a characin found in the tropical waters of Amazon Basin of Brazil and Peru) require different care and water chemistry.

We obtained 200 juvenile rummy nose tetra from a tropical fish supplier (Cichlid Exchange, Portland, OR) for use in experiments. We kept tetras in two 40 gallon home acrylic tanks (100 to each tank), heated at a constant 27 ± 0.5°C. Local tap water was very hard and unsuitable for tetras. Therefore, the water used for tetras was a 1:3 mixture of dechlorinated tap water to reverse osmosis water. We kept the water free of nitrites and low nitrates (< 10), low GH (100 ± 20 ppm), and a pH=6.8 ± 0.2. At all times, the water was circulated with a filter (Fluval 406) and aerated with small air pump. Tetras were 3.4 ± 0.5 cm in body length.

The golden shiners *N. crysoleucas* were acquired from Anderson Minnows. We kept approximately 500 juvenile shiners in three 30 gallon home tanks (~ 150 in each tank) using de-chlorinated, aerated, and filtered tap-water kept at 21°C. Shiners were 5.3 ± 0.5 cm in body length.

Both species of fish experienced a 12:12 hour light:dark cycle. A mixture of crushed high quality flakes and pellets (Tetra Min Flakes and Hikari brine shrimp, micro pellets, and freeze-dried blood worms) was fed to the fish 4 times daily. We performed weekly 30% water changes to keep nitrates and nitrites low (~ 20 and 0 ppm, respectively).

Before each experimental trial, fish were gently netted in their home tanks and transferred to the experimental tank. For each species, the water in the experimental tank was matched to the water chemistry of the respective species home tank. Fish were appropriately acclimatized to the water in the experimental tank.

A. Apparatus and protocol

The experimental apparatus consisted of a shallow tank, high-speed camera, and a visible light projector. A schematic and a labelled photograph of the apparatus are shown in Figures S1. The entire apparatus was enclosed in a blackout curtain during experiments to isolate the system from external visual stimuli. The experimental tank is quasi two-dimensional tank constructed out of clear acrylic. The tank has dimensions 183×102 cm and contains water (matching the water chemistry of each fish respective home tank) with a depth of 8 ± 1 cm.

Videos were captured by a high speed USB3 camera (Point Grey GS3-U3-41C6NIR-C) placed 180 cm above the experimental tank. The camera records 2048×1280px images at 30 frames per second. The tank is illuminated from beneath by two dozen 850nm infrared LEDs (CMVision-IR200) positioned to achieve a uniformly backlit image as shown in Fig. S1. The camera is equipped with a 10mm Schneider Cinegon lens and an IR filter to attenuate visible light. The camera is hardware triggered to synchronize with the projected light field.

A projector (BenQ W1070 1080P) casts the dynamic light field onto the experimental tank at 30 frames per second. The projector is positioned 226 cm above the tank. The measured light levels at the surface of the tank ranged from 10 lux (approximately twilight) to 500 lux (sunrise), corresponding to natural light levels in the fish habitat. Between replicates, fish were allowed to rest with the projector off (0.5 lux, deep twilight).

As shown in Fig. S2, the projected field consists of a single dark spot is overlaid with a noisy background which varied both spatially and temporally, identical to the method in a previous study[1]. The dark spot moves with a constant speed but the direction is random. We

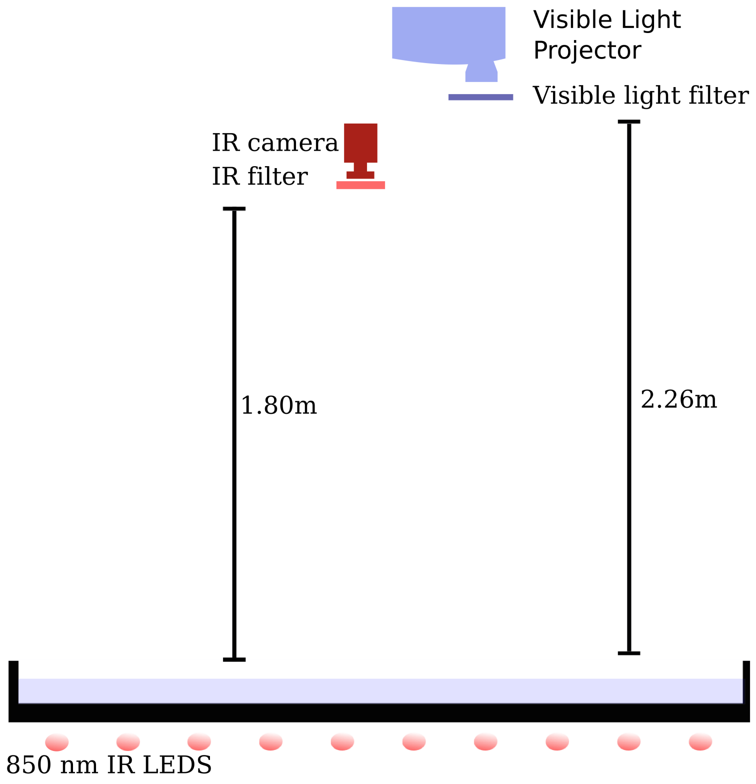

Figure S1. A schematic of the experimental apparatus. Videos of schooling events were captured via a high-speed camera mounted above the experimental arena within the aquarium. The aquarium was illuminated by an array of infrared lamps beneath the tank.

used five different seeds to generate each projected video at a medium level of environmental noise level ($\eta = 0.25$), and added a white boundary along the perimeter of the light field to discourage interaction with the sides of the arena. All light fields were displayed using psychToolbox for MATLAB at 30Hz.

Groups of N = 16, 32, 64, and 128 fish were randomly selected from the home tanks. A rotating schedule for home tanks was used to ensure that no fish were used in experiments on consecutive days. We performed all experiments between the hours of $11:00$ and $17:00$. Each experimental run lasted 5 minutes and is followed by a 10 minute rest period in a dark (0.5 lux), static, and no noise image.

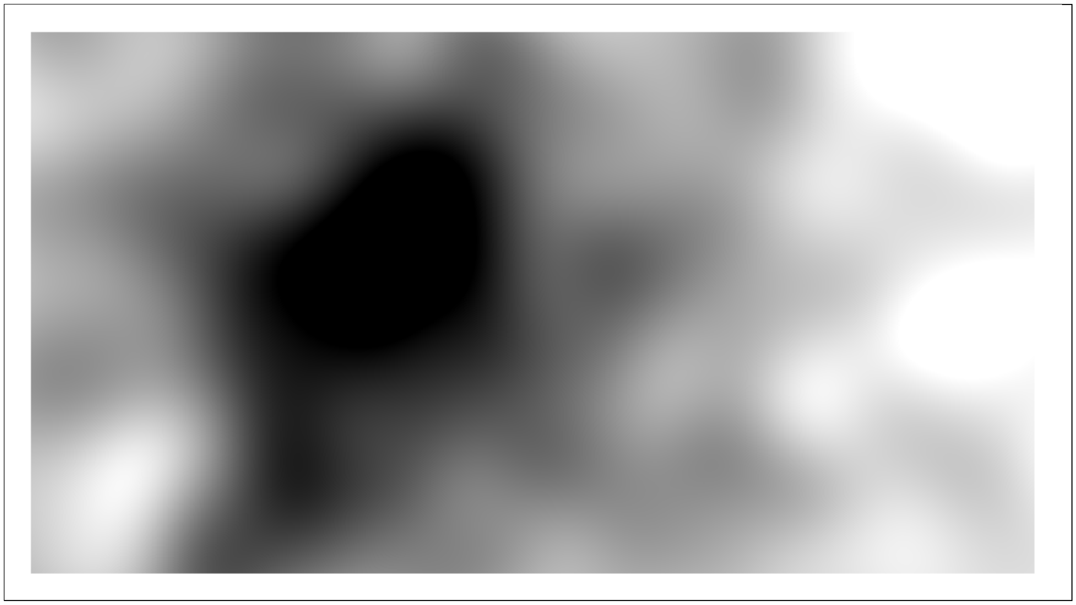

Figure S2. A sample image of the light gradient used in experiments with a white border to keep fish away from edges of the experimental tank. The thin black border is a guide to the eye and not part of the image.

B. Fish tracking software

Using image analysis, we detected all fish in each frame and link the two-dimensional positions to trajectories for each fish. In each 2D camera frame, fish are located by first applying background subtraction as shown in Fig. S3a. Since occlusions occur frequently (especially for large group sizes), simple image segmentation techniques fail to locate fish often merging two fish together as shown in the center of Fig. S3b.

Our algorithm were implemented in Python using OpenCV library[2], and followed a similar approach to SchoolTracker[3]. Fish are located using optimal pairing of line-segments detected in the background subtracted image. In each frame, the center of mass of fish are located using the paired line segments as shown in Fig. S3c.

We then track fish from frame to frame by linking the two-dimensional positions using a Kalman filter[4]. Due to the large number of fish, occlusions are frequent and the detection/tracking algorithm can fail to locate a fish over multiple frames. The tracks are spliced together by linking tracks in a four-dimensional position-velocity space[5].

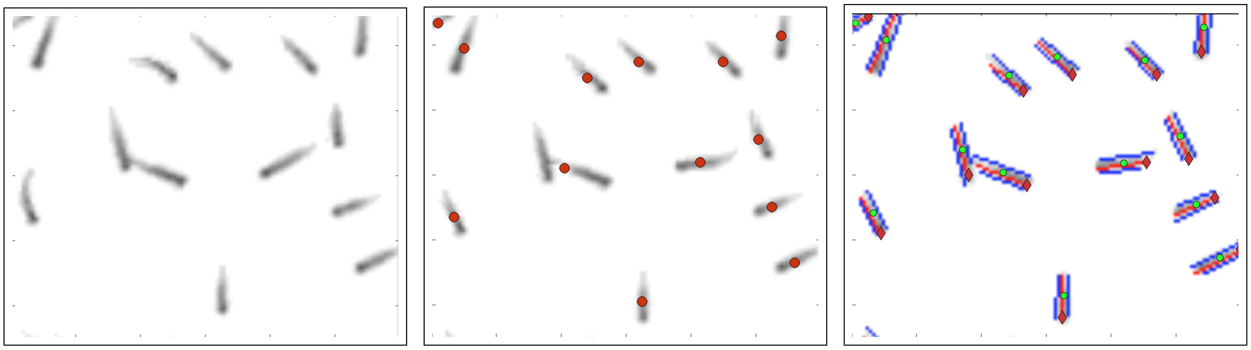

Figure S3. The background subtracted image (a) showing the fish (dark) on a white background. Standard image segmentation (b) is unable to detect fish during occlusions. In (c) we show the fish detection using our code, able to resolve occlusions.

Once the time-resolved trajectories are known, we compute velocities and accelerations by convolving the trajectories with a Gaussian smoothing and differentiating kernel[6,7]. Derivatives computed using this convolution method are less noisy than what would be obtained from a simple finite difference scheme. For the data presented here, the convolution kernel was chosen to have a standard deviation of 1.5 frames, and the position information from 11 frames was used to calculate each derivative.

C. Social vector

We calculate the social vector $\mathbf{S}$, as the sum of the unit vectors pointing from the focal fish to neighboring fish, given by,

$$\mathbf{S}_i = \sum_{j \in r_s, j \neq i} \frac{\mathbf{x}_j - \mathbf{x}_i}{|\mathbf{x}_j - \mathbf{x}_i|}. \tag{1}$$

where only neighbors are included up to a distance r_s from the focal individual. The direction of the social vector indicates the direction of social attraction and its length is a proxy for the strength of the attraction.

We calculated the environmental vector $\mathbf{G}_i$ to be the negative gradient of the light field L evaluated at the position $\mathbf{x}_i$ of each fish, $\mathbf{G}_i = -\nabla L|_{\mathbf{x}_i}$, which points in the direction of steepest descent toward darkness. The length of $\mathbf{G}$ is the rate of change of the light field in that direction.

We calculate the correlation between the direction of the corresponding vector and the direction of the fish's acceleration with the following,

$$C_{\text{social}} = \langle \widehat{\mathbf{S}}_i \cdot \widehat{\mathbf{a}}_i \rangle \tag{2}$$

$$C_{\text{environmental}} = \langle \widehat{\mathbf{G}}_i \cdot \widehat{\mathbf{a}}_i \rangle. \tag{3}$$

In Fig. S4 and Fig. S5, we show the correlations C_{social} and $C_{\text{environmental}}$ as functions of the magnitudes of the social and environmental vectors, $|\mathbf{S}|$ and $|\mathbf{G}|$, respectively. In the main text in Figure 3, we used the interaction range for the social vector to be $r_s = 7$ body lengths. In this Supplemental Information in Fig. S4 and Fig. S5, correlations C_{social} and $C_{\text{environmental}}$ are shown for $r_s = 3$, 5, and 9 for shiners and tetras, respectively.

For shiners, when the interaction range is too small, $r_s \leq 3$, while C_{social} still grows linearly with increasing magnitude of the social vector, the result is difficult to disentangle the results from effects associated with the environmental vector. However, when r_s is between 5 and 9 body lengths, we find that the results for C_{social} are with the main text.

For the tetras, in Fig. S5, we find the results of C_{social} are not sensitive to the interaction range for any $r_s \leq 9$. While we find that C_{social} is proportional to $|\mathbf{S}|$, the correlations are smaller than $C_{\text{environmental}}$, signifying that tetra's acceleration is weakly correlated with the social vector.

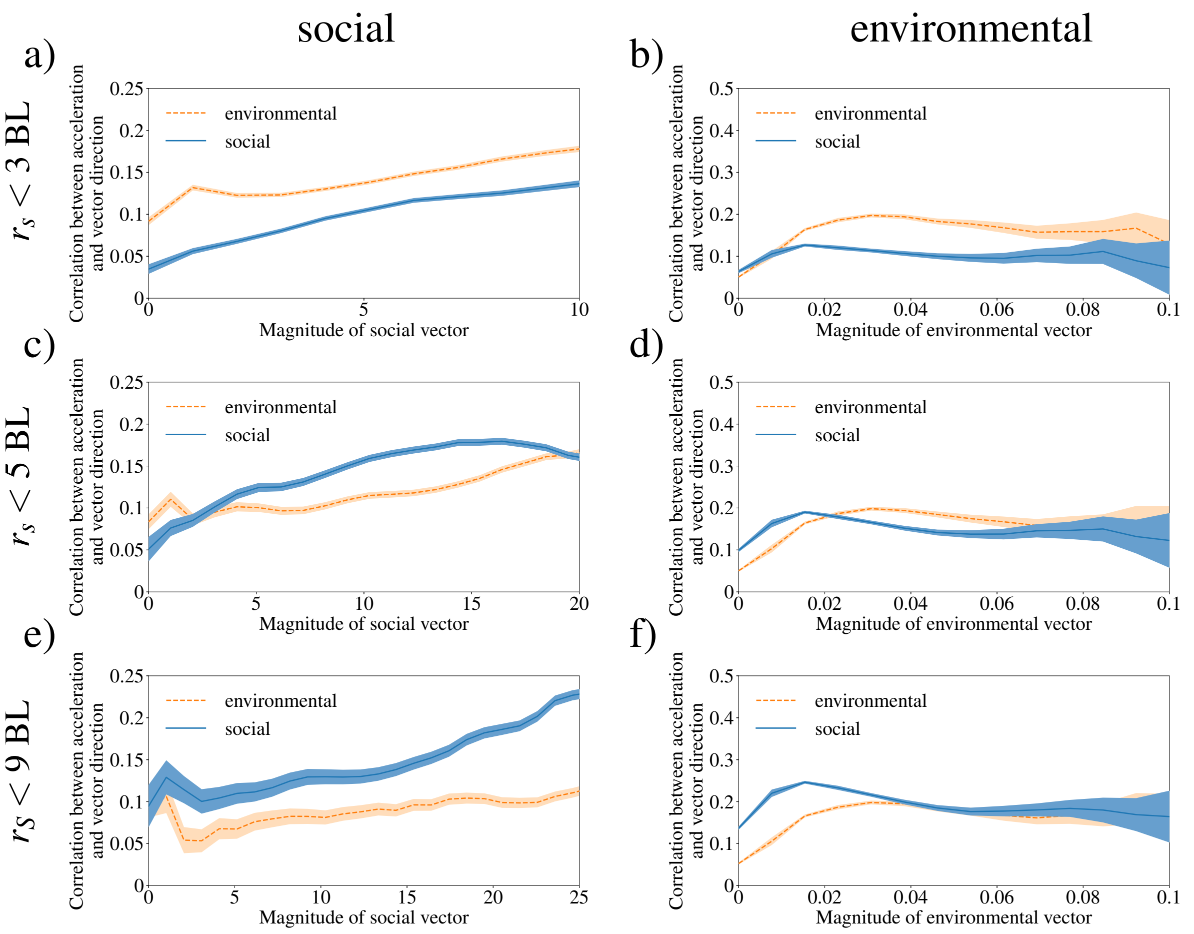

Figure S4. **Shiners.** Experimental correlations between accelerations of shiners and the social and environmental cues (y-axis) are shown as functions of the magnitude of the social (ace) and environmental (bdf) vectors (x-axis), respectively. For all subfigures, the correlation between the accelerations and social vector is dark (blue) and between the accelerations and environmental vector is light (orange). When calculating the social vector, the interaction range, r_s, is 3 body lengths (ab), 5 body lengths (cd) and 9 body lengths (ef).

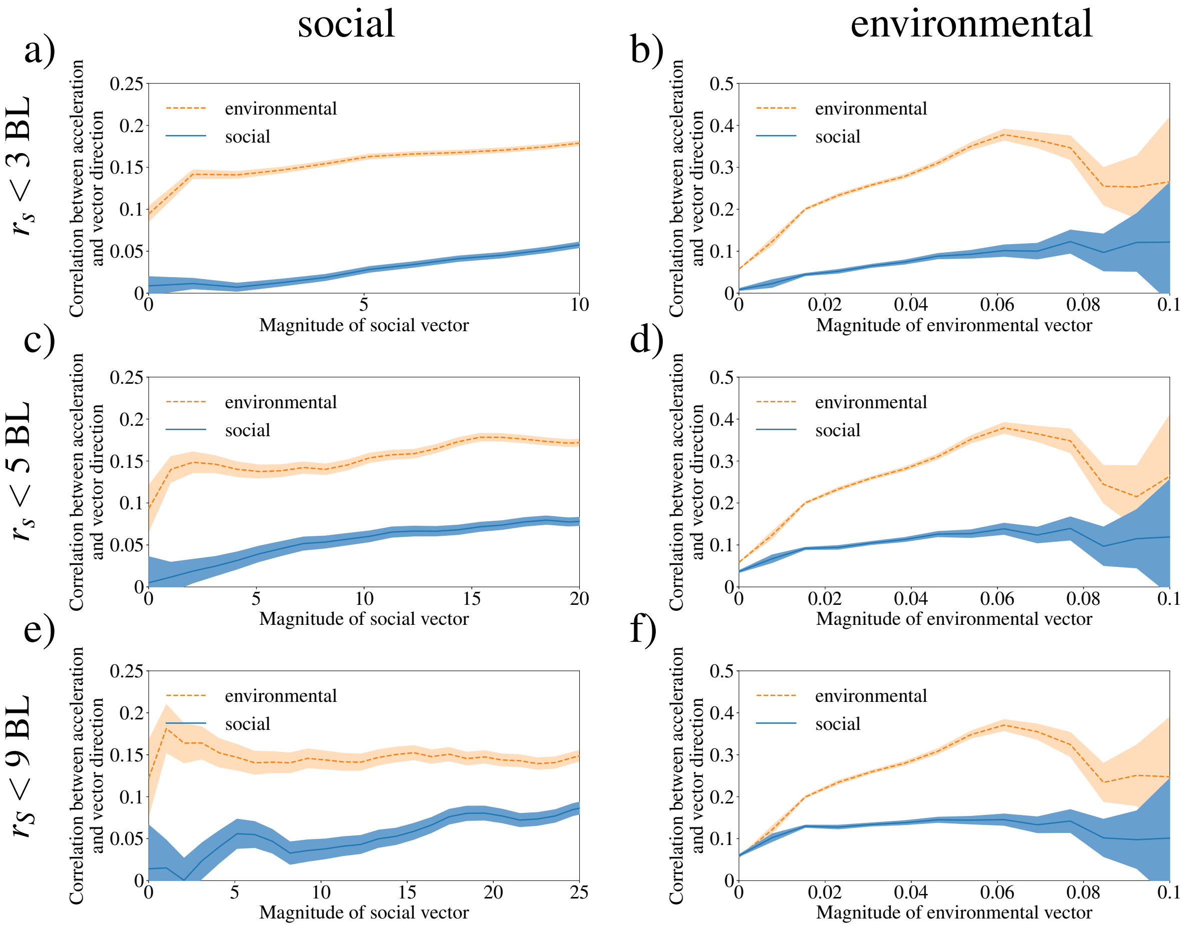

Figure S5. **Tetra.** Experimental correlations between accelerations of tetras and the social and environmental cues (y-axis) are shown as functions of the magnitude of the social (ace) and environmental (bdf) vectors (x-axis), respectively. For all subfigures, the correlation between the accelerations and social vector are dark (blue) and between the accelerations and environmental vector are light (orange). When calculating the social vector, the interaction range, r_s, is 3 body lengths (ab), 5 body lengths (cd) and 9 body lengths (ef).

II. SIMULATION

A. Model details

Our model is based on the Berdahl-Couzin model[1] where individuals interact via zonal interactions as in the Couzin model[8] and modify their velocity by slowing down in dark regions and speeding up in brighter regions. The flow of information in the model is outlined in Fig. S6. In the Berdahl-Couzin model, individuals interact based on social information using the positions of neighbours in three different zones: repulsion, orientation, and attraction.

Individuals respond to neighbours in the repulsion zone with highest priority, ignoring other social effects.

$$\mathbf{d}_{\text{repel}} = -\sum_{j\neq i}^{n_r} \frac{\mathbf{x}_j - \mathbf{x}_i}{|\mathbf{x}_j - \mathbf{x}_i|}, \tag{4}$$

where n_r is the number of neighbours in the repulsion zone.

If no neighbours are in the repulsion zone, individuals use neighbours positions and velocities to determine the direction of the orienting and attracting interactions, given by

$$\mathbf{d}_{\text{orient}} = \sum_{j\neq i}^{n_o} \frac{\mathbf{v}_j}{|\mathbf{v}_j|} \tag{5}$$

$$\mathbf{d}_{\text{attract}} = \sum_{j\neq i}^{n_a} \frac{\mathbf{x}_j - \mathbf{x}_i}{|\mathbf{x}_j - \mathbf{x}_i|}, \tag{6}$$

where n_o and n_a are the number of individuals in the orientation and attraction zones, respectively. The social direction is either the repulsive direction or the sum of the interactions from the orientation and attraction zones,

$$\mathbf{d}_{\text{social}} = \begin{cases} \mathbf{d}_{\text{repel}}, & \text{if } n_r \geq 1 \\ \mathbf{d}_{\text{orient}} + \mathbf{d}_{\text{attract}}, & \text{otherwise.} \end{cases} \tag{7}$$

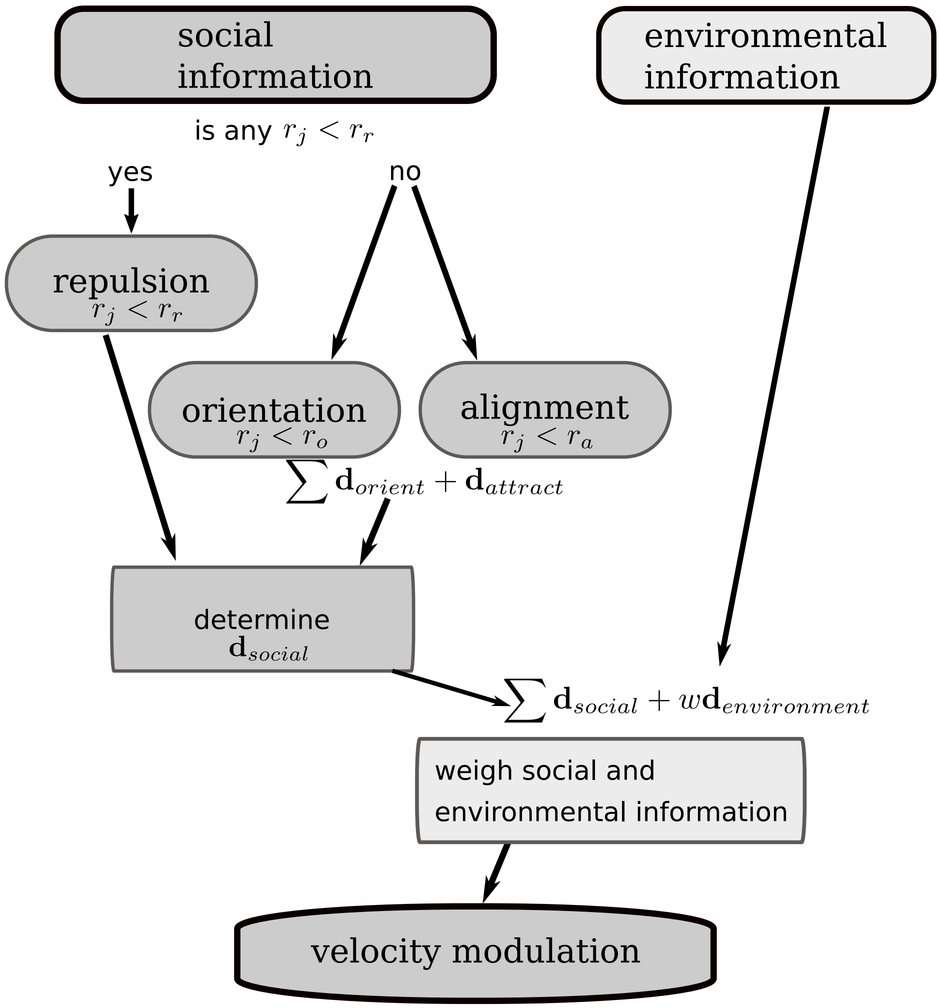

Figure S6. Outline of the simulation algorithm. The direction due to social and environmental information are computed separately and normalised. The direction of the individual is given by the sum of the social direction and the weighted environmental direction. The final velocity is the product of this direction with the light modulated speed.

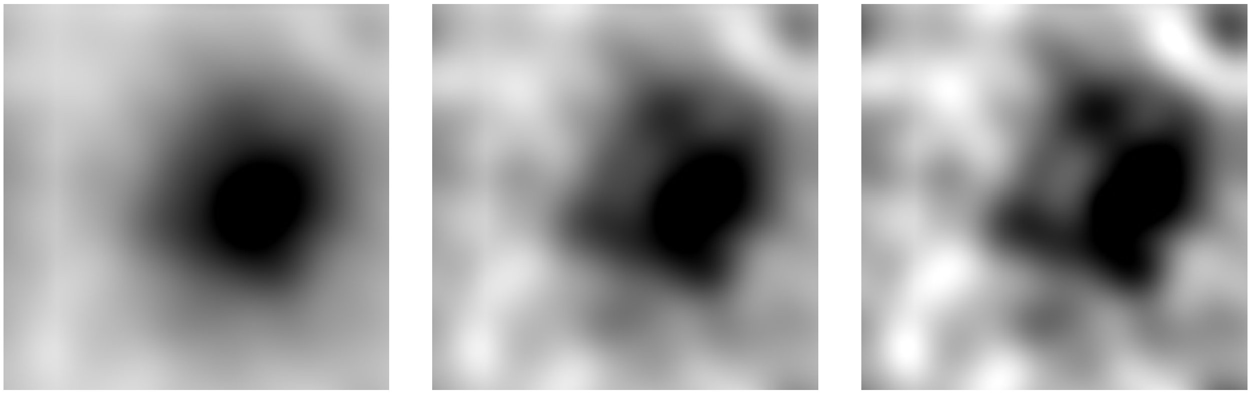

Figure S7. From left to right, sample noise image for noise scale $\eta = 0.1, 0.25$, and 0.40.

In the Berdahl-Couzin model, this direction is normalised and multiplied by the speed of the individual which is based on the local light intensity, where individuals swim slower in darker regions,

We adapt this model to include an individual environmental sensing of the light field gradient, given by

$$\mathbf{d}_{\text{environmental}} = -\nabla L, \tag{8}$$

which is normalised to determine the direction, $\widehat{\mathbf{d}}_{\text{environmental}}$, due to the environmental light field L.

We then weight the social and environmental sensing, where the direction of individuals is given by the following,

$$\widehat{\mathbf{d}} = \frac{\widehat{\mathbf{d}}_{\text{social}} + w\ \widehat{\mathbf{d}}_{\text{environmental}}}{|\widehat{\mathbf{d}}_{\text{social}} + w\ \widehat{\mathbf{d}}_{\text{environmental}}|}, \tag{9}$$

where w is the tunable weighting parameter.

We ignore very small gradients where magnitude of the environmental gradient $|\mathbf{d}_{\text{gradient}}| < 0.0001$, to avoid singularities inside the dark region. Note, that in the dark region, interactions then default directly to social interactions only.

The velocity of the individual is then given by the product of the overall weighted and normalised direction and the speed given by the light intensity modulated speed,

$$\mathbf{v} = s\ \widehat{\mathbf{d}}, \tag{10}$$

where s is the speed.

For all our experiments, we use the same simulation parameters for the interaction zones and the light field characteristics as the Berdahl-Couzin model[1], which are detailed in Table S1. Sample images for noise levels $\eta = 0.1, 0.25$, and 0.40 are shown in Fig. S7.

B. Parameters for Berdahl-Couzin model for tetras

In the main text, all numerical results use $r_o = 3$ and $r_a = 5.5$, which was chosen to closely match values accepted for shiners[1]. We now investigate the parameters for tetras using the Berdahl-Couzin (or our model with no gradient sensing ability $w = 0$). In Fig. S8, we show the root mean square error in group performance Ψ between experimental data for tetras and Berdahl-Couzin simulations using $r_o, r_a \in [0, 7.5]$ evaluated at each 0.5 body lengths. The results of the simulation are averaged over twenty random seeds. While there is a region in the parameter space which minimises the root mean square error for the tetras near $r_o = 1.0$ and $r_a = 3.5$, the root mean square error for tetras is several times larger than the error for shiners previously reported[1] (Berdahl et. al., Supplemental Information).

We then re-run our simulations and analyse the results parallel to the main text Fig. 6ab using Berdahl-Couzin parameters ($r_o = 1.0$, $r_a = 3.5$). As shown in Fig. S9a, the nearest neighbour distance d_{nn} is larger for small group sizes due to the smaller interaction ranges used in the simulation for tetras ($r_o = 1.0$, $r_a = 3.5$) compared to used in the main text ($r_o = 3.0$, $r_a = 5.5$). We find $\widetilde{w}_{\mathrm{min,d}} \approx 68.1$, as the weight which minimises d_{nn} for all N. Using $\widetilde{w}_{\mathrm{min,d}}$, we plot the results of the simulated group performance for tetras in Fig. S9b and find the agreement between the experimentally measured group performance and that calculated using the model is not as good as reported in the main text.

To further investigate how the model fits our experimental data, we find the weight $\widetilde{w}_{min,\Psi}$ which minimises the root mean square error, RMSE=$\langle(\Psi_w - \Psi_{\mathrm{tetra}})^2\rangle_N$, using $r_o = 1.0$ and $r_a = 3.5$. We find the weight which minimises the RMSE $\widetilde{w}_{min,\Psi} \approx 21.5$ is smaller than the weight which minimised the nearest neighbor distance $\widetilde{w}_{min,d} \approx 68.1$. Note, that using the $r_o = 3.0$ and $r_a = 5.5$ as in the main text, we find that $\widetilde{w}_{min,d} = \widetilde{w}_{min,\Psi}$.

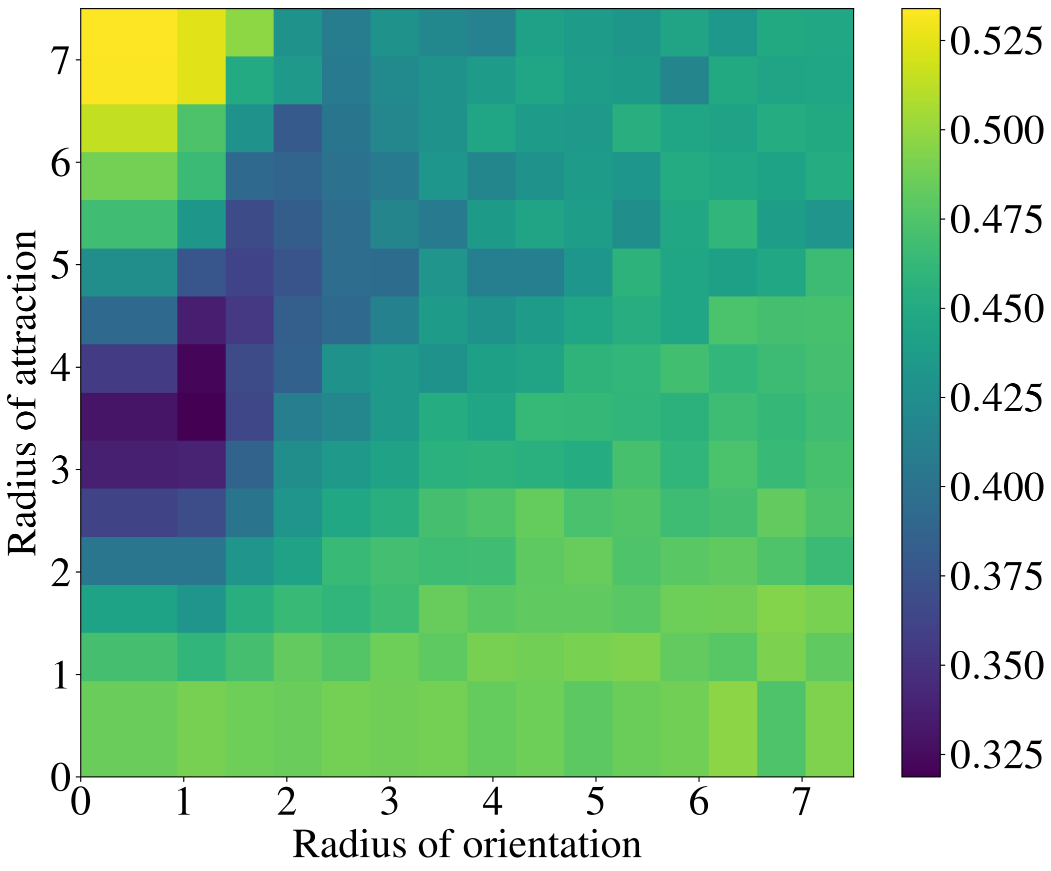

Figure S8. **Tetra**: Root mean square error between group performance Ψ of the Berdahl-Couzin zonal model[1,8] and experimental data for tetras. For the parameters in the band of dark purple area, the Berdahl-Couzin model is more closely matching the tetras.

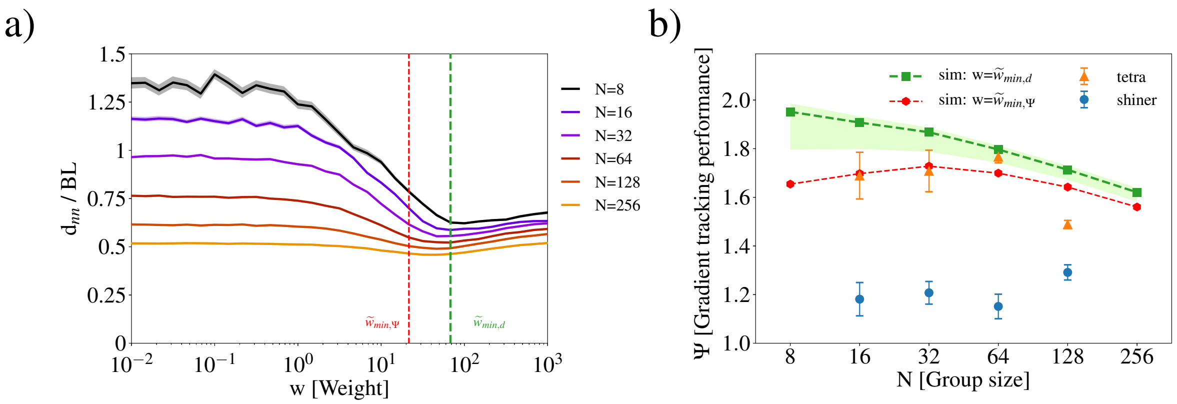

Figure S9. Nearest neighbour distance of simulated schools (using $r_0 = 1.0$ and $r_a = 3.5$) is shown as a function of the weight w for group sizes N. The thick dashed vertical line is the weight $\widetilde{w}_{\rm min}$ which minimises d_{nn} for all N. The thin dashed vertical line is the weight $w = \widetilde{w}_{min,\Psi}$ which minimises the root mean square error between the model and the tetra data. (b) Gradient tracking performance of the numerical results for $\widetilde{w}_{\rm min,d} \approx 21.5$ (dark green squares), where the shaded region shows the range of Ψ corresponding to simulated weights from $w \approx 46$ to 100. The Ψ using the weight $\widetilde{w}_{min,\Psi}$ is marked with red hexagons. The experimental results are overlaid for the rummy nose tetras (orange triangles) and shiners (blue circles).

The results presented in Fig. S9ab use the Berdahl-Couzin parameters ($r_o = 1.0$, $r_a = 3.5$), which minimised the RMSE in performance between the tetras and the model using $w = 0$. To fully parameterise the model, optimising the model to better predict the behaviour for the tetras, the RMSE between our experimental results and numerical simulations must be optimised over the entire parameter space (r_o, r_a, w). Given that the RMSE using the weight $\widetilde{w}_{min,d}$ is smaller using $r_o = 3.0$ and $r_a = 5.5$ (RMSE=0.075) than for $r_o = 1.0$ and $r_a = 3.5$ (RMSE=0.235), the best fit parameters are likely to be nearer to the former values which used in the text. Moreover, running simulations over the entire parameter space (r_o, r_a, w) for several seeds and group sizes N is very computationally expensive.

Finally, given the good agreement between group performance of the tetras and the simulations used in the main text with $r_o = 3$, $r_a = 5.5$ and $w = \widetilde{w}_{min,d} = 31.6$, our results represent proof-of-principle that tetras can sense gradients and moderate their environmental information with social information.

Table S1. Parameter values for experiment and simulation in the main text. These simulation values were constant for all the simulations performed except notified in the text.

Parameter	*Domain*		
	Image [px]	Arena [cm]	Simulation[cm]
Tank Length	960	183	140
Tank Width	540	102	140
Decay Length of the Patch (D)	200	38.1	38.5
Speed of the patch cm/s	30	5.71	5.71
Body Length	-	5	5
Radius of repulsion	-	-	2.5
Radius of alignment	-	-	15
Radius of attraction	-	-	27.5
Max swim speed	-	25 cm/s	25cm/s
Min swim speed	-	1 cm/s	1cm/s
Direction update Rate	-	-	8/s
Max turning rate	-	-	1.75radians/update
Rotational noise	-	-	0.01
Min Gradient sensing	-	-	0.0001

REFERENCES

[1] A. Berdahl, C. J. Torney, C. C. Ioannou, J. J. Faria, and I. D. Couzin, Science **339**, 574 (2013).

[2] *Open Source Computer Vision Library (OpenCV)*.

[3] S. B. Rosenthal, C. R. Twomey, A. T. Hartnett, H. S. Wu, and I. D. Couzin, Proceedings of the National Academy of Sciences of the United States of America **112**, 4690 (2015).

[4] G. Welch and G. Bishop, Tech. Rep. (2006).

[5] H. Xu, Measurement Science and Technology **19**, 075105 (2008).

[6] N. Mordant, A. Crawford, and E. Bodenschatz, Physica D: Nonlinear Phenomena **193**, 245 (2004), 0303003.

[7] N. Ouellette and J. Gollub, Physical Review Letters **99**, 1 (2007).

[8] I. D. Couzin, J. Krause, R. James, G. D. Ruxton, and N. R. Franks, Journal of Theoretical Biology **218**, 1 (2002).
